# Notfall-Neuropädiatrie – Der arteriell ischämische Schlaganfall als einer der zeitkritischsten Notfälle bei Kindern und Jugendlichen

**DOI:** 10.1007/s00115-021-01252-4

**Published:** 2022-01-24

**Authors:** Lucia Gerstl, M. Olivieri, F. Heinen, C. Bidlingmaier, A. S. Schroeder, K. Reiter, F. Hoffmann, K. Kurnik, T. Liebig, C. G. Trumm, N. A. Haas, A. Jakob, I. Borggraefe

**Affiliations:** 1 Abteilung für Pädiatrische Neurologie, Entwicklungsneurologie und Sozialpädiatrie, LMU Zentrum für Entwicklung und komplex chronisch kranke Kinder – iSPZ Hauner, Kinderklinik und Kinderpoliklinik im Dr. von Haunerschen Kinderspital, LMU Klinikum München, Campus Innenstadt, Lindwurmstr. 4, 80337 München, Deutschland; 2 Abteilung für Pädiatrische Hämostaseologie, Kinderklinik und Kinderpoliklinik im Dr. von Haunerschen Kinderspital, LMU Klinikum München, Campus Innenstadt, München, Deutschland; 3 Abteilung für Kinderintensivmedizin und Notfallmedizin, Kinderklinik und Kinderpoliklinik im Dr. von Haunerschen Kinderspital, LMU Klinikum München, Campus Innenstadt, München, Deutschland; 4 Institut für Diagnostische und Interventionelle Neuroradiologie, LMU Klinikum München, Campus Großhadern, München, Deutschland; 5 Abteilung Kinderkardiologie und Pädiatrische Intensivmedizin, LMU Klinikum München, Campus Großhadern, München, Deutschland

**Keywords:** Ätiologie, Diagnostik, Klinische Präsentation, Sekundärprophylaxe, Rezidivrisiko, Etiology, Diagnostics, Clinical presentation, Secondary prophylaxis, Recurrence risk

## Abstract

Der arteriell ischämische Schlaganfall im Kindes- und Jugendalter gehört zu den zeitkritischsten Notfällen in der Pädiatrie. Dennoch wird er häufig mit einer oft prognostisch relevanten Zeitverzögerung diagnostiziert. Gründe dafür liegen neben der geringen Awareness auch in der zuweilen unspezifischen klinischen Präsentation mit einer herausfordernden Breite kritischer Differenzialdiagnosen sowie in der Fläche noch wenig verzahnter Akutversorgungsstrukturen. Gleichwohl zeigen grundsätzlich die beim Erwachsenen etablierten Revaskularisationsstrategien auch beim Kind ihre möglichen, zum Teil spektakulären Erfolge. Es gilt also, diese nach Möglichkeit auch den betroffenen Kindern zur Verfügung zu stellen, auch wenn hier derzeit ein nicht annähernd vergleichbarer Grad an Evidenz erreicht ist. Postakut ist die ätiologische Aufarbeitung durch die größere Bandbreite zu bedenkender Risikofaktoren besonders komplex, muss aber in der Lage sein, das individuelle Risikoprofil mit Sekundärprophylaxe, Rezidivrisiko und Outcome präzise zu identifizieren. Die Langzeitbetreuung im multiprofessionellen, interdisziplinären Team muss die biopsychosozialen Aspekte des Kindes in seiner jeweiligen Entwicklungsphase berücksichtigen und damit eine bestmögliche Integration des Kindes in sein soziales und schulisches, später berufliches Umfeld realisieren.

## Abstract (en)

Arterial ischemic stroke in childhood and adolescence is one of the most time-critical emergencies in pediatrics. Nevertheless, it is often diagnosed with a considerable time delay which may be associated with low awareness, the sometimes nonspecific clinical presentation with a wide variety of differential diagnoses, and less established ‘acute care structures’. The revascularisation strategies in adult stroke care are also potential and promising treatment options for children, even if available evidence is still limited. In the post-acute phase, the etiological work-up is complex due to the multitude of risk factors to be considered. But it is essential to identify each child’s individual risk profile as it determines secondary prevention, risk of recurrence and outcome. Long-term care in a multiprofessional, interdisciplinary team must take into account the bio-psycho-social aspects to integrate the child into its social and educational, and later professional environment.

Für den Schlaganfall im Erwachsenenalter liegen evidenzbasierte Diagnostikalgorithmen und Therapieempfehlungen vor, neue Versorgungsmodelle (neurovaskuläre Netzwerke) sind erfolgreich und die Stroke-Units flächendeckend etabliert. Diese breiten, dynamisch sich positiv entwickelnden Erfahrungen aus der Erwachsenenneurologie lassen sich jedoch weder „einfach“ noch „unreflektiert“ auf das pädiatrische Kollektiv – mit anderer Biologie und einem sich entwickelnden Gehirn – übertragen. Zu groß sind die Unterschiede bezogen auf die Ätiologie und zu spezifisch sind die Anforderungen an optimierte pädiatrisch-spezialisierte Versorgungsstrukturen, die es in den kommenden Jahren unter Einbeziehung digitaler/telemedizinsicher Möglichkeiten versorgungsgerecht in Deutschland aufzubauen gilt.

## Hintergrund

Der arteriell ischämische Schlaganfall (AIS) im Kindes- und Jugendalter (> 28. Lebenstag bis 18 Jahre) ist mit einer Inzidenz von 1–8/100.000 Kinder pro Jahr selten und unverändert mit hoher Morbidität und Mortalität assoziiert [19, 36, 43]. Er ist einer der zeitkritischsten Notfälle in der Pädiatrie und zählt weltweit zu den 10 häufigsten Todesursachen im Kindesalter. Er betrifft prinzipiell alle Altersstufen, epidemiologische Daten zeigen einen Häufigkeitsgipfel bei Säuglingen und Vorschulkindern und einen erneuten Inzidenzanstieg bei Jugendlichen [23]. Die Ursache für die unterschiedlichen Inzidenzen in den verschiedenen Altersstufen konnte bislang noch nicht eindeutig geklärt werden [23, 43]. Im Kleinkindalter könnte das häufigere Auftreten von (banalen) Infektionen ein erhöhtes Risiko für einen AIS bedingen [17, 60].

## Klinik

Unabhängig vom Alter präsentieren sich die meisten Kinder mit einem akut auftretenden fokal neurologischen Defizit, die Leitsymptome wie die akute Hemiparese, faziale Parese und Sprachstörung finden sich auch im Kindesalter: Diese Symptome werden im FAST(„face, arm, speech, time“)-Test erkannt [23, 25]. Der FAST-Test zeigte in einer Studie von Yock-Corrales et al. bei pädiatrischen Patienten eine Sensitivität von 76 % (vergleichbar mit der Sensitivität im Erwachsenenalter), wobei diese bei Infarkten in der vorderen Zirkulation wesentlich höher lag als bei Infarkten in der hinteren Zirkulation (88 % vs. 50 %) [61].

Bei Erwachsenen erhöht der be(„balance, eyes“)FAST-Test die Sensitivität bei Infarkten der hinteren Zirkulation noch einmal deutlich, sodass empfohlen wird, im klinischen Setting auch bei Kindern und Jugendlichen bevorzugt den beFAST-Test anzuwenden [4].

Die klinische Präsentation des AIS ist umso unspezifischer, je jünger das Kind ist

Trotz klarer Leitsymptome wird die Diagnose des kindlichen Schlaganfalls durch das Auftreten weiterer unspezifischer Symptome erschwert [23, 39]. Dazu zählen insbesondere bei Säuglingen und kleinen Kindern das Auftreten von Krampfanfällen (bis zu 50 %) und bei Schulkindern das Auftreten von (andere neurologische Symptome begleitende, in der Regel nicht isolierte) Kopfschmerzen. Auch „stotternde“ Verläufe mit wechselnder Symptomatik sind bekannt und treten insbesondere bei Kindern mit Arteriopathien auf. Grundsätzlich gilt: Die klinische Präsentation eines Kinders mit AIS ist umso unspezifischer, je jünger das Kind ist.

## „Stroke mimics“

Eine besondere Herausforderung in der raschen Diagnose des AIS stellen im Kindesalter die gut bekannten und wesentlich häufigeren „stroke mimics“ dar. In einer Studie war bei Kinder mit akut fokal neurologischem Defizit lediglich bei 7 % der Kinder ein Schlaganfall für die akute Neurologie ursächlich – im Vergleich zu über 70 % bei Erwachsenen [37]. Bei Kindern waren als Ursache die Migräne, Krampfanfälle und die idiopathische Fazialisparese führend [38, 39]. Die Entwicklung eines spezifischen Früherkennungstools – „childhood stroke recognition tool“ – mit der Möglichkeit der Abgrenzung zu den häufigen Differenzialdiagnosen ist Ziel verschiedener Studien, bislang aber noch nicht erfolgreich.

Wichtig: Die Diagnose (und Ausschlussdiagnose) eines Schlaganfalls gelingt ausschließlich mit der Bildgebung, die bei jeder akuten fokalen Neurologie („acute brain attack“) umgehend veranlasst werden muss.

## MERCS-Pocketcard – be FAST!

**Figure 1:**
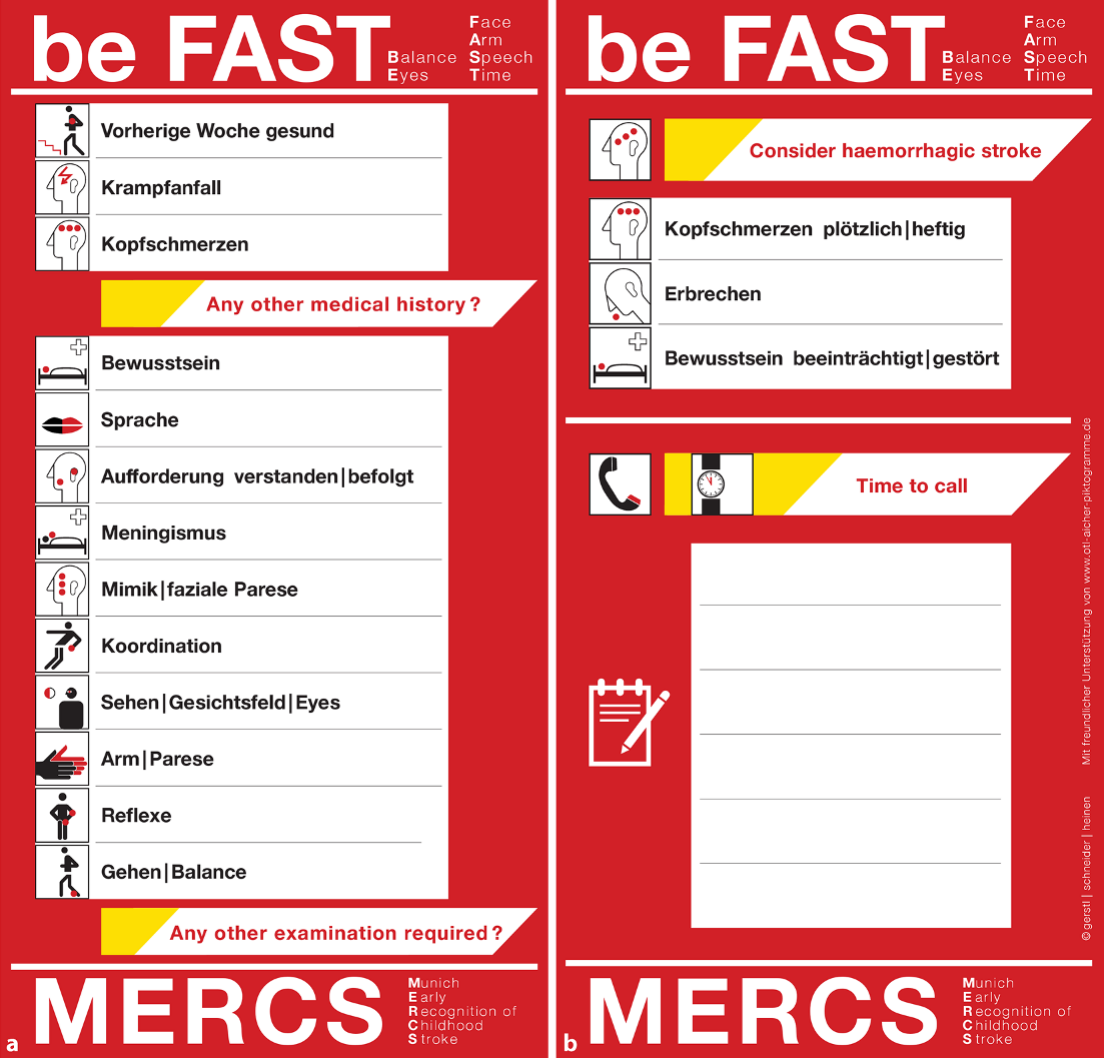

Die Pediatric Stroke Arbeitsgruppe des LMU Klinikums in München hat die MERCS(Munich Early Recognition of Childhood Stroke)-Pocketcard entwickelt (Abb. 1). Neben dem beFAST-Test werden die wesentlichen Punkte einer kinderneurologischen Untersuchung in der Notfallsituation aufgeführt und um relevante klinische Information zum ischämischen und hämorrhagischen Schlaganfall ergänzt. Auch wenn mit dieser Karte keine zweifelsfreie klinische Unterscheidung zwischen „stroke“ und „stroke mimic“ getroffen werden kann, so hat sie sich im klinischen Alltag sehr bewährt und leistet einen wesentlichen Beitrag zur Awareness des kindlichen Schlaganfalls und frühzeitigen Alarmierung der Notfallkette.

## Risikofaktoren/Ätiologie

Die Hauptrisikofaktoren für einen Schlaganfall im Erwachsenenalter wie z. B. arterielle Hypertonie, Rauchen, Vorhofflimmern, Diabetes mellitus spielen im Kindesalter keine relevante Rolle. Zu den Risikofaktoren beim kindlichen Schlaganfall zählen Arteriopathien, kardiale Ursachen, Infektionen, angeborene thrombogene Blutgerinnungsstörungen, hämatologisch-onkologische Erkrankungen, genetische Prädispositionen, metabolische Ursachen und Bindegewebserkrankungen [20, 21, 23, 24, 43, 57]. Eine Übersicht möglicher Risikofaktoren findet sich in Tab. 1.

**Table 1:** 

Ursachen und Risikofaktoren	
Arteriopathien	Fokale zerebrale Arteriopathie (FCA)
	Para-/postinfektiöse Vaskulitis
	Moyamoya-Angiopathie
	Dissektion der extra- und intrakraniellen Hirngefäße
	Primäre ZNS-Vaskulitis
	Genetische Prädisposition (s. unten)
	Fibromuskuläre Dysplasie
	Systemischer Lupus erythematodes
Kardiovaskuläre Ursachen	Angeborene/erworbene Herzfehler
	Postoperativ Fremdkörper linksseitig (Patches etc.)
	Persistierendes Foramen ovale (PFO)
	Intrapulmonale Shunts (arteriovenöse Malformation)
	Endokarditis
	Schlechte linksventrikuläre (LV-)Funktion (Myokarditis, Kardiomyopathie)
	Tachykarde Herzrhythmusstörungen
	Kardiochirurgischer Eingriff/Herzkatheteruntersuchung
	Mechanische Kreislaufunterstützung
	Selten: kardiale Tumoren (Vorhofmyxom)
	Gefäßverengungen postoperativ
	Inflammatorische Gefäßveränderungen (Kawasaki, Takayasu)
Koagulopathien	Protein-C-Mangel
	Protein-S-Mangel
	Prothrombinmutation (G20210A)
	Faktor-V-Leiden-Mutation (G1691A)
	Antithrombinmangel
	Lipoprotein-a-Erhöhung
	Faktor-VIII-Erhöhung
	Hyperhomozysteinämie
	Antiphospholipidantikörpersyndrom
Hämato-onkologische Ursachen	Sichelzellkrankheit
	Hämolytische Anämie, Hämoglobinopathien
	Eisenmangelanämie
Metabolische Ursachen	Mitochondriopathie
	CDG(„congenital disorders of glycosylation“)-Syndrom
	Homozystinurie
Genetische Prädisposition	Trisomie 21
	Neurofibromatose Typ 1
	PHACE(„posterior fossa anomalies, hemangioma, arterial anomalies, cardiac anomalies and eye anomalies“)-Syndrom
	Alagille-Syndrom
	Mutation: ACTA2, ADA2 (*CECR1*-Gen), SMAD3, FOX1, Col4A1, Col4A2, RNF213
Bindegewebserkrankungen	Ehrlers-Danlos-Syndrom
	Marfan-Syndrom
Infektionen	Varizella-zoster-Virus („post varicella vasculopathy“)
	*Borrelia burgdorferi*
	*Mycoplasma pneumoniae*
	Enteroviren
	Parvoviren
	Herpes-simplex-Virus (HSV)
	Epstein-Barr-Virus (EBV)
	Meningitis durch Pneumokokken, *Mycobacterium tuberculosis*
	Allgemein: Sepsis, Dehydratation
Medikamente/Therapien	Kontrazeptiva
	L‑Asparaginase
	Strahlentherapie
Sonstige	Zerebrovaskuläre Anomalien (Anmerkung: Hauptrisikofaktor für hämorrhagischen Schlaganfall)
	Hirntumor
	Zustand nach neurochirurgischem Eingriff
	Trauma/Schädel-Hirn-Trauma
	Migräne (Rolle nicht abschließend geklärt)

Der Schlaganfall im Kindesalter ist als „multiple risk disease bekannt“, d. h. bei einem relevanten Anteil der betroffenen Kinder liegen mindestens zwei Risikofaktoren vor [23, 35]. Das Vorliegen mehrerer Risikofaktoren scheint mit einem schlechteren Outcome assoziiert zu sein.

Bei ca. 10–20 % der Kinder findet sich trotz umfassender Diagnostik keiner der bislang bekannten Risikofaktoren (kryptogener Schlaganfall).

Im Folgenden sollen einige (häufige) Risikofaktoren näher ausgeführt werden.

### Arteriopathien

Arteriopathien stellen mit bis zu 50 % den häufigsten Risikofaktor für einen AIS im Kindesalter dar und spielen darüber hinaus eine Rolle für das Auftreten von Schlaganfallrezidiven [29, 43].

Zu den wichtigsten Arteriopathien zählen die fokale zerebrale Arteriopathie, die Dissektion und die Moyamoya-Angiopathie. Mit den Fortschritten in der genetischen Diagnostik werden auch zunehmend Arteriopathien auf der Basis eines genetischen Hintergrundes diagnostiziert [47].

#### Fokale zerebrale Arteriopathie

**Figure 2:**
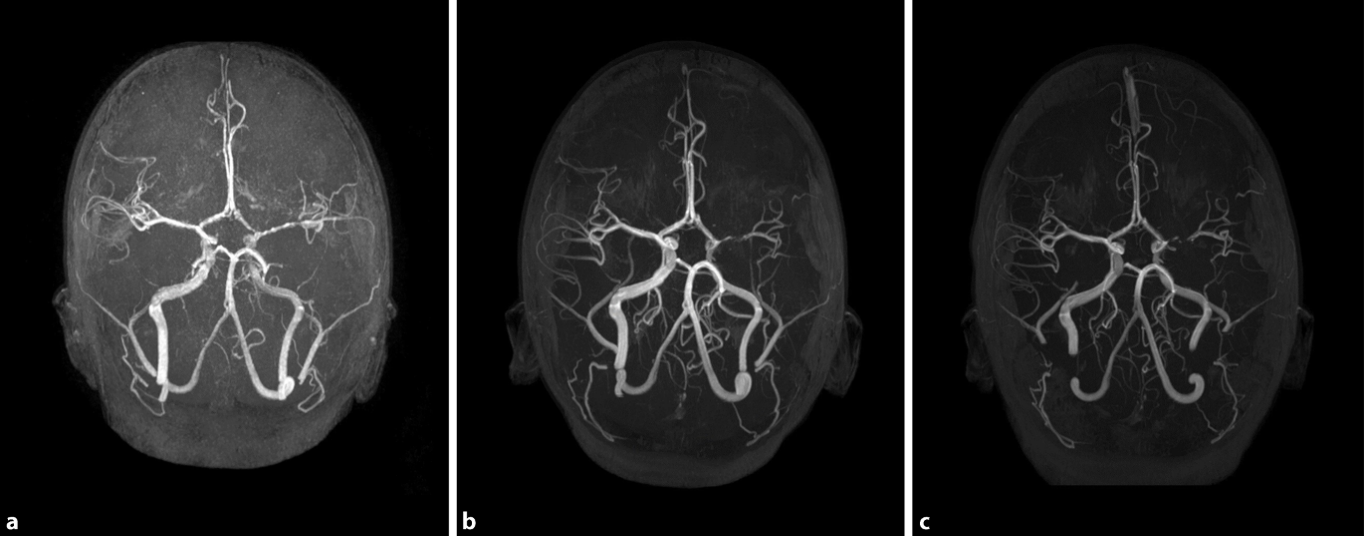

Die fokale zerebrale Arteriopathie (FCA; Synonyme: transiente zerebrale Arteriopathie, transiente fokale Arteriopathie) ist die häufigste Arteriopathie und tritt meistens unilateral im Bereich der distalen A. carotis interna (ICA), proximalen A. cerebri media (MCA) und proximalen A. cerebri anterior (ACA) auf, wobei sie nur selten die hintere Strombahn betrifft [8, 9, 18, 33]. Die Pathophysiologie dieser meist perlschnurartig anmutenden fokalen Stenosierung der Gefäße ist nicht eindeutig geklärt. Vermutet wird eine post-/parainfektiöse Schwellung der Gefäßwand mit Stenosierung, die in den ersten Tagen bis Wochen rasch progredient sein kann; in dieser Zeit ist das Rezidivirisiko am höchsten (Abb. 2). Der Verlauf ist in der Regel nach 6 bis 12 Monaten selbstlimitierend.

**Figure 3:**
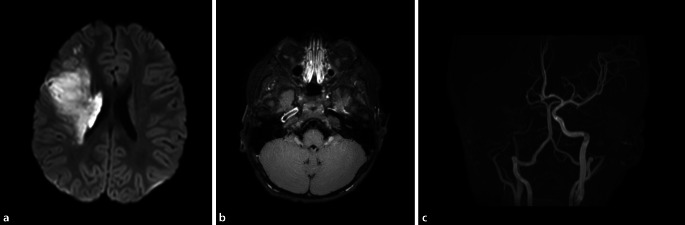

Als bekannteste Ursache für eine FCA gilt die (Post‑)Varizellen-Arteriopathie (Varicella-zoster-Virus[VZV]-Arteriopathie), die auch noch Wochen bis Monate nach einer akuten VZV-Infektion auftreten kann (Abb. 3, [3, 34]). Aber auch andere Erreger wie z. B. Herpes-simplex-Virus oder *Borrelia burgdorferi* können zu einer FCA führen.

Mit der PASTA-Studie (Paediatric Arteriopathy Steroid Aspirin Trial), einer internationalen multizentrischen randomisiert-kontrollierten Studie, wird aktuell ein möglicher Benefit einer zusätzlich zur Standardtherapie mit Aspirin eingesetzten Steroidtherapie beim Vorliegen einer FCA untersucht (PI: M. Steinlin, Bern).

#### Zerebrovaskuläre Dissektion

Dissektionen finden sich bei Kindern mit Schlaganfall relativ häufig. Sie entstehen meist nach Traumata (auch Bagatelltraumata, Autounfall – Gurt) während Sport und Spiel [40, 51]. Klinisch führend sind heftige, plötzlich auftretende einseitige Schmerzen im Kopf, Gesicht oder Halsbereich, die dem eigentlichen ischämischen Infarkt vorausgehen.

Als prädisponierende Faktoren gelten Bindegewebserkrankungen und inflammatorisch veränderte Gefäßwände. Die Dissektionen finden sich sowohl extrakraniell als auch intrazerebral, eine Abgrenzung zur FCA kann bei letzterem manchmal schwierig sein [11].

#### Moyamoya

Die Moyamoya-Angiopathie ist charakterisiert durch einen wahrscheinlich inflammatorisch getriggerten Gefäßwandumbau (v. a. der distalen ICA oder MCA) mit progredienter Stenosierung bis hin zum Verschluss und im Verlauf mit der Ausbildung der charakteristischen zahlreichen leptomenigealen Kollateralen, deren angiographischer Aspekt dem Krankheitsbild den Namen gab („puffy smoke“ – Moyamoya; [32]).

Unterschieden wird die Moyamoya-Erkrankung (primär, idipoathisch) vom Moyamoya-Syndrom (sekundär), bei dem die Gefäßveränderungen im Rahmen einer prädisponierenden Grunderkrankung auftreten (u. a. bei Trisomie 21, Neurofibromatose oder Sichelzellkrankheit). Transiente ischämische Attacken – im Kindesalter ansonsten selten – treten bei einem Großteil der Kinder mit Moyamoya-Angiopathie auf (ca. 70 %), häufig finden sich bei Diagnosestellung auch bereits ältere Infarkte [2].

#### Arteriopathien mit genetischer Prädisposition

Verschiedene anamnestische, klinische oder bildgebende Hinweise indizieren eine genetische Abklärung. Dazu gehören u. a. das Auftreten hämorrhagischer und ischämischer Infarkte (beim Kind oder in der Familie), charakteristische bildgebende Befunde wie gestreckte, intrazerebrale Gefäße bei der *ACTA2*-Mutation oder das Vorhandensein pathognomonischer Symptomkombinationen, wie z. B. wiederholte Fieberschübe mit Livedo racemosa und Hepatosplenomegalie bei der ADA2-Defizienz. [47]. Mit zunehmender Häufigkeit genetischer Diagnostik wächst auch das Wissen um mögliche prädisponierende genetische Faktoren. Erwähnt seien hier auch die zunehmenden Erkenntnisse zur *RNF213*-Mutation und ihrer phänotypischen Bandbreite [52].

### Infektionen

Infektionen scheinen ein wichtiger Risikofaktor für den kindlichen Schlaganfall zu sein [13, 17, 60]. Auch nach sog. „minor infections“ wie z. B. Infektionen der oberen Luftwege oder akute Otitis media zeigte sich in der VIPS-Studie (Vascular Effets of Infections in Pediatric Stroke Study) das Schlaganfallrisiko um das bis zu 6‑Fache erhöht. Bei der bakteriellen oder tuberkulösen Meningitis/Meningoenzephalitis stellt der Schlaganfall eine bekannte Komplikation dar. Die Rolle verschiedener Pathogene bei der Entstehung der para‑/postinfektiösen vaskulitischen Veränderungen (Stichwort: FCA, Dissektion) wurde unter „Arteriopathien“ bereits beschrieben.

Welche Rolle eine Infektion mit SARS-CoV‑2 („severe acute respiratory syndrome coronavirus type 2“) bei der Entstehung zerebrovaskulärer Ereignisse bei Kindern spielen könnte, ist derzeit noch nicht geklärt [6]. Es wird zum jetzigen Zeitpunkt empfohlen, alle Kinder mit akutem Schlaganfall auf SARS-CoV‑2 zu testen.

### Kardiale Ursachen

Etwa 20–30 % der Schlaganfälle im Kindesalter haben eine kardiovaskuläre embolische Ursache [12, 23, 30, 43]. Zu den typischen kardialen Gründen zählen angeborene Herzfehler mit linksseitigen Klappenvitien und v. a. alle Vitien mit kardialer Zyanose (inkl. postoperativ, z. B. Fontanzirkulation), bei denen Thromben ohne die Filterfunktion der Lunge eine Emboliequelle darstellen können. Zu dieser Gruppe zählen auch intrapulmonale Shunts.

Etwa 20–30 % der Schlaganfälle im Kindesalter haben eine kardiovaskuläre embolische Ursache

Frisch postoperativ können intrakardiale Patches oder andere Fremdoberflächen Gründe für eine Thrombenbildung darstellen. Gleiches gilt für künstliche oder auch für native Klappen im Rahmen einer Endokarditis. Prozedurale Komplikationen mit Luft- oder Thrombembolie und sehr selten anderen biologischen Materialien (z. B. Kalk) können im Rahmen von Operationen bzw. Herzkatheteruntersuchungen auftreten, entweder als Komplikation oder als thrombotisches Ereignis aufgrund einer inadäquaten Heparinisierung (Antikoagulation). Eine erheblich reduzierte Funktion des Systemventrikels (Kardiomyopathie, Myokarditis) begünstigt intra(ventrikuläre)-kardiale Thromben, insbesondere, wenn zusätzliche Risikofaktoren vorliegen (z. B. Non-compaction-Kardiomyopathie).

Eine weitere Gruppe an Risikopatienten stellen die Kinder und Jugendlichen dar, bei welchen extrakorporale Verfahren (extrakorporale Membranoxygenierung [ECMO], extrakorporale Lungenunterstützung [ECLA], links „ventricular assist device“ [LVAD] etc.) zum Einsatz kommen.

Eine seltene Ursache sind intrakardiale Tumoren (Vorhofmyxom) oder Tumoren mit intrakardialer Ausdehnung (z. B. Neuroblastom), die entweder direkt embolisieren können oder als Basis für thrombotische Auflagerungen dienen. Vorhofrhythmusstörungen oder tachykardieinduzierte Kardiomyopathien mit intrakardialen Thromben (Vorhofohr) sind ebenfalls selten.

Eine weitere Ursache findet sich in Stenosierungen der herznahen Gefäße, die entweder nativ (Aortenbogenanomalien) oder postoperativ (Abgangsstenosen der Kopf-Hals-Gefäße nach Kardiochirurgie) oder im Rahmen von Katheterinterventionen (intravaskuläre Stents) auftreten können. Diese Stenosierungen treten auch bei typischen Vaskulitiden (z. B. Kawasaki etc.) auf oder können sich sehr selten im Rahmen der hereditären Aortopathien (z. B. Ehlers-Danlos‑, Loeys-Dietz‑, Marfan-Syndrom) als seltene Komplikation präsentieren.

Auch im (Kindes- und) Jugendalter spielt die sog. „paradoxe Embolie“ über ein persistierendes Foramen ovale (PFO) eine bedeutsame Rolle beim sog. „cryptogenic stroke“. Dies gilt insbesondere dann, wenn ein Vorhofseptumaneurysma vorliegt und weitere prädisponierende Faktoren, die eine venöse Thrombose begünstigen (orale Antikonzeption, prothrombotische Risikofaktoren, venöse Malformationen [May-Thurner-Syndrom]), oder andere Risikofaktoren vorliegen (Thrombus am rechtsatrialen Katheter, Schrittmacherelektrode), bei denen eine passagere (Pressen, Husten) oder eine permanente pulmonale Hypertonie (Lungenembolie, primäre pulmonale Hypertension etc.) einen Rechts-links-Shunt über das PFO begünstigen. Hier ist die Indikation zum Verschluss gegeben [26, 55].

### Prothrombotische Risikofaktoren

Die Rolle angeborener und erworbener prothrombotischer Risikofaktoren in der Ätiologie des kindlichen Schlaganfalls ist gut beschrieben [5, 31, 48]. Die Risikoerhöhung variiert je nach Risikofaktor, deren isoliertem oder kombiniertem Vorliegen sowie der Koexistienz weiterer, nicht hämostaseologischer Risikofaktoren. Als „etablierte“ prothrombotische Risikofaktoren gelten: Antithrombinmangel, Protein-C-Mangel, Protein-S-Mangel, Faktor-V-Leiden-Mutation (G1691A), Prothrombinmutation (G20210A), Anticardiolipin-Antikörper, Lupus-Antikoagulans, Lipoprotein-(a)-Erhöhung und hohe Faktor-VIII-Spiegel.

## Diagnostik

### Bildgebung in der Akutphase

Die Diagnose wird mit der Bildgebung gestellt. Goldstandard in der Diagnostik des kindlichen Schlaganfalls ist die kraniale Magnetresonanztomographie (cMRT; [42, 45, 53, 58]). Vorteile liegen zum einen in der Möglichkeit einer frühen Infarkterkennung, einer gegenüber der Computertomographie (CT) besseren Beurteilbarkeit der hinteren Schädelgrube und den Abgrenzungsmöglichkeiten zu den wesentlich häufigeren „stroke mimics“. Für die initiale Diagnostik ist ein kurzes MRT-Protokoll über 10–20 min ausreichend, es sollte folgende Sequenzen beinhalten.

- diffusionsgewichtete Sequenz (DWI) mit Diffusionskoeffizienten-Karte (ADC map),
- Gradientenechosequenz (GRE) oder suseptibilitätsgewichtete Sequenz (SWI),
- 3D time-of-flight (TOF) MR-Angiographie der zerebralen und zervikalen Gefäße,
- Fluid-attenuated inversion recovery Sequenz (FLAIR) > 1. Lebensjahr; T2-gewichtete Sequenz < 1. Lebensjahr

Zum Nachweis einer zerebralen Ischämie ist es für die akute Therapieentscheidung in der Regel ratsam, eine kontrastmittelgestützte MR-Angiographie (bessere Beurteilbarkeit einer zugrunde liegenden Gefäßpathologie) durchzuführen. Bei Diskrepanz von Klinik und Bildbefund kann eine MR-Perfusion helfen, das Vorliegen und Ausmaß einer Perfusionsminderung zu erfassen.

Ein besonderer Fokus muss auf die Gefäßwanddarstellung gelegt werden

Im Hinblick auf die relativ hohe Inzidenz von Arteriopathien beim kindlichen Schlaganfall muss spätestens in der weiterführenden MRT-Diagnostik ein besonderer Fokus auf die Gefäßwanddarstellung gelegt werden.

Ist die Durchführung eines cMRTs nicht zeitnah (bis spätestens 60 min) nach Klinikeintritt möglich, soll eine cCT inklusive cCT-Angiographie durchgeführt werden, die dann im Verlauf (empfohlen innerhalb 24–48 h) um eine dedizierte MRT-Diagnostik ergänzt wird. Eine cCT wird zudem prioritär beim bewusstseinsgestörten Kind zum raschen Ausschluss einer intrakraniellen Blutung, Raumforderung oder Liquorzirkulationsstörung eingesetzt.

### Weitere Diagnostik

Zur Basisdiagnostik bei jedem Kind zählen neben der kardiologischen Abklärung eine laborchemische, infektiologische und immunologische Diagnostik sowie Untersuchungen auf prothrombotische Risikofaktoren. Bei einigen Kindern kann auch eine genetische Untersuchung sinnvoll sein [14, 44]. Wie umfangreich die ätiologische Abklärung sein muss, ist u. a. abhängig von der (Familien‑)Anamnese und den bildgebenden Befunden. Das heißt, nicht jedes Kind braucht alles, aber alle Kinder brauchen viel.

## Therapie

### Thrombolyse und mechanische Thrombektomie

Prospektive klinische Studien zum Einsatz der Revaskularisationstherapien (Lysetherapie und mechanische Thrombektomie [MT]) im Kindesalter fehlen. Die Seltenheit der Erkrankung, aber v. a. die späte Diagnosestellung lassen die Durchführung prospektiver, randomisierter Studien auch im internationalen, multizentrischen Setting zum jetzigen Zeitpunkt wenig realisierbar erscheinen (vgl. Abbruch der TIPS-Studie (Thrombolysis in Pediatric Stroke Study) zur Lysetherapie 2013; [54]).

Eine Revaskularisation kann auch bei Kindern eine vielversprechende Therapieoption sein

Zur Thrombolyse wie auch zur MT wurden in den letzten Jahren mehrere retrospektive Fallserien und Kohortenstudien publiziert, in denen auch bei (kleinen) Kindern eine Revaskularisationstherapie sicher, erfolgreich und mit gutem Outcome durchgeführt werden konnte [1, 7, 16, 22, 50, 56]. Zu bedenken sind jedoch weiterhin die noch begrenzten Fallzahlen in den Studien. So wurde in der fallzahlstärksten Studie (Save ChildS Study von Sporns et al.) 73 Kinder in 27 internationalen Stroke-Zentren behandelt [56].

Zum jetzigen Zeitpunkt sollte bei einem Großgefäßverschluss und/oder Mismatch (Diffusion-Perfusion oder Diffusion-klinisches Bild) im MRT eine medikamentöse und/oder mechanische Rekanalisationstherapie nach sorgfältiger Nutzen-Risiko-Abwägung im multidisziplinären Team erwogen werden.

Zusammenfassend können diese beiden Therapieoptionen derzeit nur als Off-label-Therapien und somit im Sinne eines individuellen Heilversuchs eingesetzt werden. Ihr Einsatz soll daher den spezialisierten pädiatrischen Schlaganfallzentren vorbehalten sein, die über die notwendige Expertise und Multidisziplinarität verfügen.

### Antithrombotische Therapie

Bei fehlender Indikation für eine Thrombolyse oder MT sollte schnellstmöglich eine Antikoagulation mit unfraktioniertem/niedermolekularem Heparin bzw. Thrombozytenaggregationshemmung mit Acetylsalicylsäure (ASS) begonnen werden, eine Evidenz für oder wider Heparin oder ASS in der Akutphase fehlt [10, 14, 44, 46]. Im Verlauf kann die Therapie und anschließende Sekundärprophylaxe je nach Ursache des Schlaganfalls umgestellt werden. Direkte orale Antikoagulanzien (DOAK) sind zur Therapie des Schlaganfalls im Kindesalter nicht zugelassen und sollten außerhalb von Studien nicht eingesetzt werden.

Die Dauer der Sekundärprophylaxe ist abhängig von der zugrunde liegenden Ätiologie, dem individuellen Rezidivrisiko und den Befunden in der Bildgebung und beträgt bei den meisten Kindern mindestens 2 Jahre.

### Steroide/immunsuppressive Therapie

Bei Patienten mit primärer Klein- oder Großgefäßvaskulitis des Zentralnervensystems haben Steroide und ggf. weitere Immunsuppressiva einen festen Stellenwert [58]. Inwieweit Steroide auch das Outcome bei Patienten mit FCA verbessern könnten, wird aktuell in der PASTA-Studie (siehe oben) untersucht.

### Funktionelle Therapie

In der Postakutphase gilt es, funktionelle medizinische Therapien (Physio‑, Ergo- und Sprachtherapie), moderne Übungskonzepte wie Robotik, Botulinumneurotoxin (BoNT) und Virtual Reality aufeinander abzustimmen und die Beratung/Aufklärung der Familien über das Erkrankungsbild und die individuelle Prognose bzw. die weitere Betreuung im Team zu leisten.

## Rezidivrisiko/Outcome

Verglichen mit dem sehr geringen Rezidivrisiko nach perinatalem Stroke (ca. 2 %), liegt das Rezidivrisiko nach Schlaganfall im Kindes- und Jugendalter zwischen 6–40 % und wird primär nicht durch das Alter, sondern durch die zugrunde liegende Ätiologie und das individuelle Risikoprofil eines jeden Kindes bestimmt [28, 57, 59].

Kinder ohne oder mit nur einem nachgewiesenen (bislang bekannten) Risikofaktor haben ein wesentlich geringeres Rezidivrisiko als Kinder mit mehreren Risikofaktoren. Bei stenosierenden Arteriopathien wie der Moyamoya-Angiopathie oder der Sichelzellkrankheit ist das Rezidivrisiko besonders hoch. Motorische Einschränkungen (v. a. spastische Hemiparese) zeigen ca. zwei Drittel der Kinder nach Schlaganfall, auch die Post-stroke-Epilepsie ist häufig [15, 41].

Die gesundheitsassoziierte Lebensqualität ist bei Kindern mit AIS signifikant niedriger als bei Gleichaltrigen [27, 49]. Aufgrund dieser schweren Auswirkungen eines Schlaganfalls für die betroffenen Kinder und ihre Familien ist eine langfristige interdisziplinäre multiprofessionelle biopsychosoziale Betreuung wichtig. Es gilt, die Bedarfe der Kinder nach einem Schlaganfall in den unterschiedlichen Lebensphasen zu kennen und interdisziplinär ein bestmögliches, personalisiertes Therapiekonzept zu erarbeiten.

## Ausblick

Grundvoraussetzung für eine verbesserte Versorgung von Kindern mit Schlaganfall sind neben der stärkeren Implementierung der Kinderneurologie als Notfalldisziplin und der Definition und Verwendung klinikinterner „standard operating procedures“ (SOPs) auch – überregional/flächendeckend – der Aufbau pädiatrischer neurovaskulärer Netzwerke und die Nutzung telemedizinischer Möglichkeiten in enger Zusammenarbeit mit *allen* Fächern der Neuromedizin.

Verschiedenen Initiativen – national wie international – ist es gelungen, das Kind mit Schlaganfall mehr ins Bewusstsein zu rücken und Schritt für Schritt Awareness für und Wissen über den Schlaganfall im Kindesalter zu erhöhen. Beispielhaft genannt seien hier:

- das 2015 gegründete „Deutsche Netzwerk Pediatric Stroke“ (Lead: LMU München, Dachgesellschaft: Gesellschaft für Neuropädiatrie, GNP), ein multidisziplinäres Netzwerk, dem mittlerweile mehr als 35 Akut- und Rehabilitationskliniken sowie Vertreter aus Österreich, der Schweiz und den Niederlanden angehören,
- das Schlaganfall-Kinderlotsen-Projekt der Stiftung Deutsche Schlaganfall-Hilfe,
- die International Pediatric Stroke Organization, IPSO (gegründet 2019).

## Fazit für die Praxis

- Ein Schlaganfall im Kindesalter („childhood stroke“) kann in jeder Altersstufe auftreten.
- Leitsymptom ist auch beim Kind die akute, fokale Neurologie (be FAST!).
- „Childhood stroke“ ist selten, „stroke mimics“ sind häufig. Die Diagnose wird nur mit der Notfallbildgebung gestellt (Goldstandard: kraniale Magnetresonanztomographie).
- Die Ursache ist häufig multifaktoriell, die Bandbreite der abzuklärenden Risikofaktoren groß: Arteriopathien, kardiale Erkrankungen und Koagulopathien stehen an erster Stelle.
- Revaskularisationstherapien sind auch beim Kind vielversprechende Therapieoptionen in der Akutphase. Ihr Einsatz als Off-label-Therapien bedarf der Expertise spezialisierter Pediatric-Stroke-Zentren in ihrer eingespielten Interdisziplinarität über Fachgrenzen hinaus.
- Bestehende Versorgungsstrukturen in der Akutphase für Kinder mit Schlaganfall sind unzureichend: Ein wichtiger Ansatz muss hier der Aufbau pädiatrischer neurovaskulärer Netzwerke sein, die alle Möglichkeiten digitaler Medizin vorhalten und flächendeckend nutzen.
